# Transpinal and Transcortical Stimulation Alter Corticospinal Excitability and Increase Spinal Output

**DOI:** 10.1371/journal.pone.0102313

**Published:** 2014-07-09

**Authors:** Maria Knikou

**Affiliations:** 1 The Graduate Center, City University of New York, New York, New York, United States of America; 2 Departments of Physical Therapy & Neuroscience, College of Staten Island/CUNY, Staten Island, New York, United States of America; 3 Sensory Motor Performance Program, Rehabilitation Institute of Chicago, Chicago, Illinois, United States of America; 4 Department of Physical Medicine and Rehabilitation, Northwestern University Feinberg School of Medicine, Chicago, Illinois, United States of America; University of Sydney, Australia

## Abstract

The objective of this study was to assess changes in corticospinal excitability and spinal output following noninvasive transpinal and transcortical stimulation in humans. The size of the motor evoked potentials (MEPs), induced by transcranial magnetic stimulation (TMS) and recorded from the right plantar flexor and extensor muscles, was assessed following transcutaneous electric stimulation of the spine (tsESS) over the thoracolumbar region at conditioning-test (C-T) intervals that ranged from negative 50 to positive 50 ms. The size of the transpinal evoked potentials (TEPs), induced by tsESS and recorded from the right and left plantar flexor and extensor muscles, was assessed following TMS over the left primary motor cortex at 0.7 and at 1.1× MEP resting threshold at C-T intervals that ranged from negative 50 to positive 50 ms. The recruitment curves of MEPs and TEPs had a similar shape, and statistically significant differences between the sigmoid function parameters of MEPs and TEPs were not found. Anodal tsESS resulted in early MEP depression followed by long-latency MEP facilitation of both ankle plantar flexors and extensors. TEPs of ankle plantar flexors and extensors were increased regardless TMS intensity level. Subthreshold and suprathreshold TMS induced short-latency TEP facilitation that was larger in the TEPs ipsilateral to TMS. Noninvasive transpinal stimulation affected ipsilateral and contralateral actions of corticospinal neurons, while corticocortical and corticospinal descending volleys increased TEPs in both limbs. Transpinal and transcortical stimulation is a noninvasive neuromodulation method that alters corticospinal excitability and increases motor output of multiple spinal segments in humans.

## Introduction

Movement is relayed and integrated at different levels across the neural axis. Corticospinal neurons innervate all regions of the spinal grey matter, including motoneurons, and terminate bilaterally within the ventromedial zone and contralateral within the dorsolateral zones, while some of the crossed fibers terminate also in the motor nuclei [1]. Based on the anatomical orientation of the corticospinal pathway, and that the spinal cord integrates and interprets a plethora of inputs channeled in specific neuronal pathways subserving human movement [2], [3], we hypothesized that transpinal and transcortical stimulation alters corticospinal excitability and spinal motor output in humans.

Noninvasive transcutaneous electric stimulation of the spine (tsESS) over the thoracolumbar or cervicothoracic region in healthy humans at rest induces compound action potentials in distal and proximal muscles of upper and lower limbs, termed here transpinal evoked potentials (TEPs) [4]–[8]. Transcranial magnetic stimulation (TMS) delivered at 50% of the soleus maximal motor evoked potential (MEP) increased the amplitude of the soleus tsESS-induced TEPs during voluntary plantarflexion, while at specific time delays the soleus TEPs and MEPs were summated [9], supporting further our hypothesis that transpinal stimulation alters corticospinal excitability. We have recently shown that transcutaneous magnetic or electric stimulation of the spine over the thoracolumbar or cervicothoracic region reduce significantly the amplitude of the soleus and flexor carpi radialis (FCR) H reflexes, and that the TEPs recorded from arm or leg muscles are not susceptible to homosynaptic depression, have nearly half the latency of the soleus and FCR H-reflex, and are increased upon excitation of group I afferents [10]–[12]. The concomitant depression of spinal reflex excitability by tsESS and absent frequency-dependent depression of TEPs constitutes tsESS and associated TEPs suitable for diagnostic and/or therapeutic purposes in central nervous system neurological disorders, since TEPs can bypass the pathological excitability state of spinal alpha motoneurons. However, for this to be possible, a better understanding of the neuronal pathways that tsESS is channeled in the human central nervous system is needed.

Collectively, in this study, we assessed the amplitude of MEPs upon tsESS delivered over the thoracolumbar region, and the amplitude of TEPs following subthreshold and suprathreshold TMS over the left primary motor cortex in healthy humans. We demonstrate that transpinal and transcortical stimulation alter corticospinal excitability and increase motor output of multiple spinal segments in humans.

## Materials and Methods

### Subjects

Nineteen (10 male, 9 female) adult healthy subjects between the ages of 21 and 55 (30.5±9.20; mean ± SD) participated in the study. All experimental procedures were conducted in compliance with the Declaration of Helsinki after Institutional Review Board (IRB) approval by the City University of New York (NY, USA). Each subject signed an informed consent form before study enrollment and participation. People with tooth implants, assistive hearing devices, pacemaker, history of seizures, medications known to alter central nervous system excitability, and history of neurological, muscular or psychiatric disorders were excluded from the study. To reduce TMS-related discomfort, all subjects wore a mouth guard and ear plugs during testing.

### Electromyography

Following standard skin preparation, single differential bipolar surface electromyography (EMG) electrodes (Motion Lab Systems Inc., Baton Rouge, LA, USA) were placed bilaterally on the medial gastrocnemius (MG), soleus (SOL), tibialis anterior (TA), and peroneus longus (PL) muscles, and were secured with 3M Tegaderm transparent film (3M, St. Paul, MN, USA). All EMG signals were filtered with a cut-off frequency of 20–1000 Hz (1401 plus running Spike 2; Cambridge Electronic Design, Cambridge, UK).

### Transcranial magnetic stimulation

TMS over the left primary motor cortex was delivered with single pulses using a Magstim 200^2^ stimulator (Magstim, Whitland, UK) and a double-cone coil (diameter 110 mm) placed so the current of the coil to flow from a posterior to an anterior direction, and according to procedures we have previously utilized [13]. The point where the lines between the inion and glabellum, and the left and right ear tragus met was marked on an EEG cap. The double-cone coil was placed parallel and approximately 1 cm posterior and 1 cm lateral to the left from this intersection point. With the double-cone coil held at this position, the stimulation intensity was gradually increased and the MEPs recorded from the right TA, MG, SOL, and PL muscles were observed on a digital oscilloscope (TDS 2014, Tektronix, Beaverton, OR, USA). When in three out of five consecutive TMS pulses, MEPs could not be evoked selectively in the right TA muscle at low stimulation intensities with the subject at rest, the magnetic coil was moved by few mm and the procedure was repeated. When the optimal position was identified, the TA MEP resting threshold was established and corresponded to the stimulation intensity that induced repeatable MEPs in size that had peak-to-peak amplitude approximately 50 µV [14], [15].

### Noninvasive transpinal stimulation over the thoracolumbar region

Subjects were seated semi-prone with the trunk semi-flexed on a Biodex (model 870-170 Accessory Chair, Biodex Medical Systems, Shirley, NY, USA) adjustable chair with their hips at 110°–120°, knees at 100°–125°, ankles at 90° and both feet and arms supported. Two re-usable self-adhering electrodes of 10.16×5.08 cm (cathode; Model EP84169, UniPatch, Wabasha, MA), connected to function as a single electrode, were placed on the left and right iliac crests [10], [11]. The Thoracic 10 vertebra was identified via palpation, and a monopolar stainless-steel circular handheld electrode was used to determine the most optimal stimulation site. This site corresponded to the one that at low stimulation intensities TEPs were present in most or all of the ankle muscles. When TEPs were not evoked at high stimulation intensities, the monopolar electrode moved by one or two intervertebral spaces and the procedure was repeated. When the optimal stimulation site was identified, a self-adhering electrode of 10.16×5.08 cm (same as the cathodes) was placed equally between the left and right paravertebrae sides and depending on the body height of the subject it spanned from Thoracic 10 to Lumbar 4 vertebrae levels. The anode electrode was held under constant pressure throughout the experiment and maintained via pre-wrap and athletic wrap. The anode and cathode electrodes were connected to a constant current stimulator (DS7A, Digitimer, Hertfordshire, UK), that was triggered by an analog-to-digital acquisition system with customized scripts written in Spike 2 with single pulses of 1-ms duration. The stimulation intensity during which TEPs in the leg muscles were first noted on the oscilloscope at the lowest stimulation intensity was termed as TEP threshold, and ranged from 43.2 to 86.7 mA (72.53±14.79; mean ± SD) across subjects. At these stimulation intensities, subjects reported no pain or discomfort, and the blood pressure was not altered.

### Experimental protocol

The neurophysiological tests described below were conducted in the morning on the same day with a 30-min resting time to ensure similar position of TMS and stimulating and recording electrodes for subjects who participated in both experiments.

#### Experiment 1

In this experiment, the behavior of MEPs recorded from the right ankle muscles in presence of tsESS over the thoracolumbar region was assessed in 14 subjects. With subjects seated semi-prone, and after cortical and spinal stimulation sites were determined, the MEP input-output (or recruitment) curve was first constructed. TMS was triggered with single pulses at 0.1 Hz and at least 80 MEPs were recorded at varying stimulation intensities. In 7 subjects, the recruitment curve from the left and right side TEPs was also constructed with single tsESS pulses delivered at 0.1 Hz. Further, in 14 subjects, the TMS intensity was adjusted at 1.2× TA MEP resting threshold, and MEPs were recorded from the right SOL, MG, TA, and PL muscles following tsESS at conditioning-test (C-T) intervals that ranged from negative 50 to positive 50 ms. A negative C-T interval denotes that tsESS was delivered after TMS, while a positive C-T interval denotes that tsESS was delivered before TMS. At each C-T interval, 10 MEPs at 0.1 Hz were randomly recorded.

#### Experiment 2

In this experiment, the behavior of TEPs recorded from the left and right leg muscles in presence and/or absence of corticospinal descending motor volleys was assessed in 14 subjects. With subjects seated semi-prone, and after cortical and spinal stimulation sites were identified, TEPs from the left and right SOL, MG, TA, and PL muscles were recorded under control conditions at 1.2× TEP threshold and following subthreshold and/or suprathreshold TMS at C-T intervals that ranged from negative 50 to positive 50 ms. A negative C-T interval denotes that TMS was delivered after tsESS, while a positive C-T interval denotes that TMS was delivered before tsESS. Subthreshold TMS intensity was based on absent MEPs in all right leg muscles, and was delivered at 0.79±0.13× TA MEP resting threshold across subjects. Suprathreshold TMS intensity was based on stable in amplitude TA MEPs evoked on the ascending portion of the recruitment curve, and was delivered at 1.17±0.11× TA MEP resting threshold across subjects. Conditioned and unconditioned TEPs were recorded randomly at the C-T intervals and TMS intensities tested. At each C-T interval, 15 TEPs at 0.1 Hz were recorded.

### Offline data analysis

All compound muscle action potentials recorded with subjects seated semi-prone were measured as the area of the rectified waveform for identical time durations. The stimulation intensities (as a percentage of the maximum stimulator output) utilized to construct the MEP recruitment curve were normalized to the intensity corresponding to the associated MEP threshold. Then, the MEP amplitude was expressed as a percentage of the associated maximal MEP amplitude, and the average normalized MEP size in steps of 0.05 multiples of MEP thresholds was estimated. The average normalized MEP size was grouped across subjects based on the normalized stimulation intensity and the overall mean was estimated.

A Boltzmann sigmoid function (equation 1) was then fitted to the normalized MEP sizes plotted against the normalized stimulation intensities [16]–[18]. The parameters in equation 1 denote the maximal MEP (MEPmax) size, the slope parameter of the function *m*, which is the inverse of the Boltzmann slope parameter *k* and reflects the gain of the function independently from the absolute magnitude of its maximum [17], the stimulus required to elicit an MEP equivalent to 50% of the MEPmax (S50), and the MEP amplitude at a given stimulus value MEP(s). The MEP slope was constrained to occur at a stimulus equivalent to S50 and was estimated based on equation 2, while the stimulus corresponding to the MEP threshold (MEPth) and to MEPmax was defined based on equations 3 and 4, respectively [17], [18]. This was done separately for MEPs recorded from the right TA, MG, SOL, and PL muscles for each input-output curve constructed in each subject. This analysis was also done for TEPs recorded from the right and left ankle plantar flexors and extensor muscles, and results were compared to those observed for MEPs so to establish similarities and/or differences on recruitment properties of MEPs and TEPs.
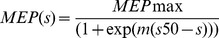
(1)

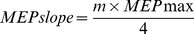
(2)

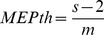
(3)

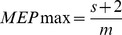
(4)


For each subject, the MEPs recorded from the right SOL, MG, TA, and PL muscles upon tsESS at different C-T intervals were expressed as a percentage of the mean amplitude of the associated unconditioned MEP. Although, the motor hot spot was located for the right TA muscle, MEPs from ankle extensors were also analyzed based on the overlap of motor cortical representation in humans [19], [20].

**Figure 1:**
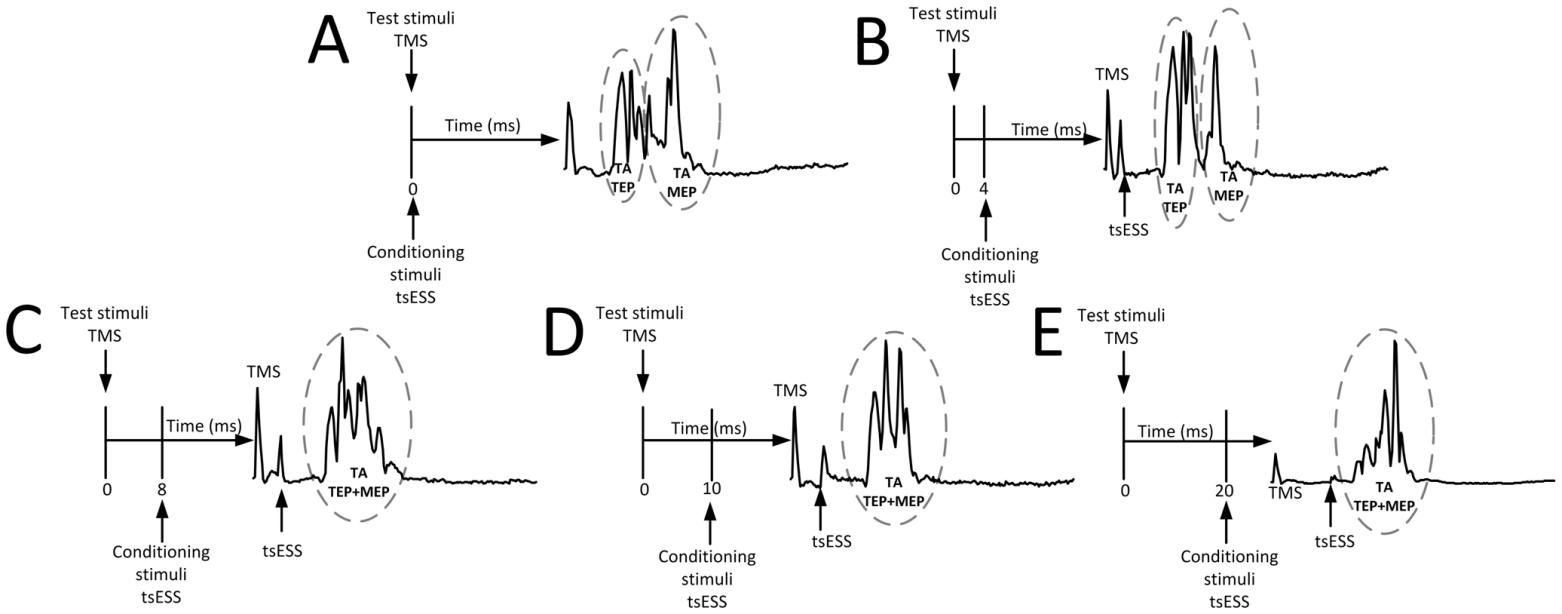
Spatial spinal summation of MEPs and TEPs. Rectified right tibialis anterior EMG following tsESS over the thoracolumbar region and TMS over the left primary motor cortex delivered at 1.3× tibialis anterior MEP resting threshold. In all paradigms TMS is the test stimuli and tsESS is the conditioning stimuli. At the conditioning-test intervals of 0 and 4 ms, the TEP following tsESS can be easily separated from the MEP based on latency and duration (**A, B**). However, at the negative C-T intervals of 8, 10, and 20 ms TEP and MEP do not occlude each other but are summated (**C, D, E**), and thus cannot be separated based on latency and duration. To counteract this neuronal phenomenon and establish the net effect of the conditioning stimulus, the control MEP values were subtracted from the conditioned TEP values and the control TEP values were subtracted from the conditioned MEP values in experiments that the conditioning stimulus was delivered at suprathreshold intensities. tsESS: transcutaneous electric stimulation of the spine. TMS: transcranial magnetic stimulation. MEP: motor evoked potential. TEP: transpinal evoked potential.

The latency of the right TA, SOL, and MG MEPs estimated based on the cumulative sum (CUSUM) technique on the rectified waveform average [10], [21], [22] was 31.62±1.97 ms, 32.62±2.31 ms, and 33.56±2.77 ms, respectively. The latency of the right TA, SOL, and MG TEPs estimated based on the CUSUM technique was 16.08±1.41 ms, 18.85±2.46 ms, and 17.83±2.18 ms, respectively and similar to those we have recently reported [11]. Based on the MEPs and TEPs latency and duration, when TMS (test stimulus) was delivered above MEP resting threshold and tsESS was the conditioning stimulus, the MEPs and TEPs were summated at the negative C-T intervals of 8, 10, and 20 ms. When the conditioning stimulus was the TMS, summation of action potentials occurred at the positive C-T intervals of 8, 10, and 20 ms. In Figure 1, a schematic illustration of the timing between test and conditioning stimuli and spatial summation between the right TA MEP and the right TA TEP from one subject is presented. Note that the right TA MEP and the right TA TEP at the negative C-T intervals of 8, 10, and 20 ms cannot be separated based on latency or duration. To counteract this neuronal phenomenon and establish the net effect of tsESS on MEPs, the associated TEP control amplitude was subtracted from the conditioned MEPs at these C-T intervals, and the resultant value was expressed as a percentage of the mean amplitude of the associated unconditioned (or control) MEP.

For each subject, the TEPs recorded from the left and right TA, MG, PL, and SOL muscles upon subthreshold and/or suprathreshold TMS at different C-T intervals were expressed as a percentage of the mean amplitude of the associated unconditioned TEP recorded at 1.2× TEP threshold. Based on the phenomenon of spatial spinal summation of MEPs and TEPs, as previously described, to establish the net effect of suprathreshold TMS on TEPs, the associated unconditioned MEP value was subtracted from the conditioned TEP value at the positive C-T intervals of 8, 10, and 20 ms, and the resultant value was expressed as percentage of the mean amplitude of the associated control TEP.

### Statistics

Mean amplitude of normalized conditioned MEPs from each subject was grouped based on the C-T interval and muscle from which it was recorded. Statistically significant differences were established with one-way analysis of variance (ANOVA) when data were normally distributed and with a Kruskal-Wallis one-way ANOVA on ranks when data were not normally distributed. When statistical significant difference was found, post hoc Bonferroni tests for multiple comparisons were conducted to establish at which C-T interval the conditioned MEP was statistically significant different. This analysis was done separately for MEPs recorded form ankle plantar flexors and extensors.

The mean amplitude of the normalized conditioned TEPs from each subject was grouped based on the C-T interval, muscle, and leg side (right/left). Statistically significant differences of the conditioned TEPs from each muscle across C-T intervals were established with one-way ANOVA, while two-way ANOVA was applied to the data to establish statistically significant differences of TEPs recorded from the right and left legs. For both tests, when statistical significant difference was found, post hoc Bonferroni t-tests for multiple comparisons were conducted. Last, a repeated measures ANOVA at 10×2×2 levels (10: C-T intervals, 2: TMS intensity, 2: right/left leg) was conducted for the TA, MG, SOL, and PL TEPs separately to establish interactions across these three different levels. Significance was set at *P*<0.05. Mean and standard error are indicated, unless otherwise stated.

## Results

### Recruitment curves of MEPs and TEPs

**Figure 2:**
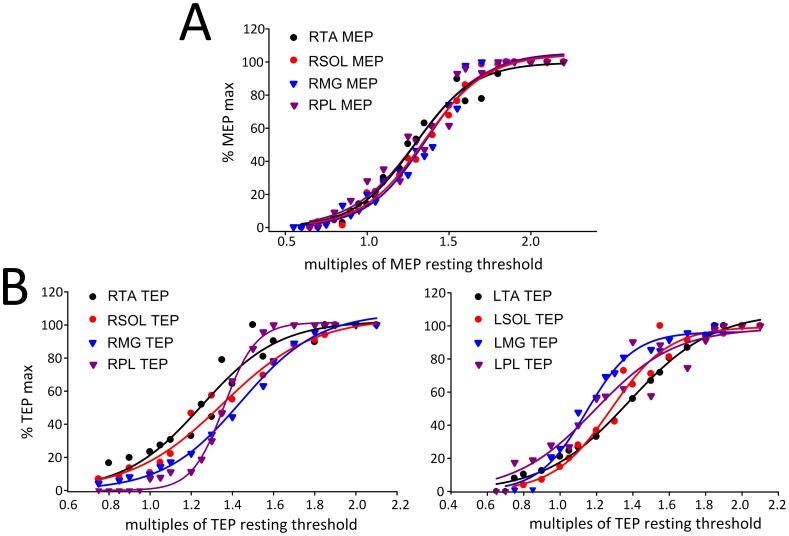
Recruitment curves of MEPs and TEPs. (**A**) MEPs recorded from 14 subjects from the right (R) TA, SOL, MG, and PL muscles while seated are plotted against the maximum stimulator output, which was normalized to the associated MEP resting threshold. (**B**) TEPs recorded from 7 subjects from the right and left TA, SOL, MG, and PL muscles while seated are plotted against the stimulation intensities, which were normalized to TEP resting threshold. TA: tibialis anterior. SOL: soleus. MG: medialis gastrocnemius. MEPs: motor evoked potentials. TEPs: transpinal evoked potentials.

In Figure 2A, the MEP recruitment curves from the right TA, SOL, MG, and PL muscles from 14 subjects are presented. MEPs were normalized to the associated maximal MEP and plotted against the percentage of the maximum stimulator output which was expressed in multiples of the associated MEP resting threshold. The recruitment curves of MEPs were not statistically significant different between TA, SOL, MG, and PL muscles (F_3_ = 1.55, *P* = 0.21; two-way ANOVA, within factors: intensity, muscle). In a similar manner, the right and left TA, SOL, MG, and PL TEP recruitment curves from 7 subjects are indicated in Figure 2B. The TEPs recruitment curves were not statistically significant different between left and right legs (F_1_ = 3.95, *P* = 0.51) or between muscles (F_3_ = 1.55, *P* = 0.2; three-way ANOVA).

The sigmoid equation described well the relationship between the amplitude of MEPs and TEPs with the normalized stimulation intensities, since the adjusted *R* ^2^ ranged from 0.94 to 0.99 across muscles and type of compound muscle action potential (Table 1). The maximum size of the MEPs and TEPs from the recruitment curve recorded from the right and/or left leg muscles along with the summarized sigmoid function parameters are presented in Table 1. No statistically significant differences were found between the sigmoid function parameters of right MEPs and right TEPs among muscles from which they were recorded (two-way ANOVA, *P*>0.05 for all parameters), and for the estimated sigmoid function parameters between TEPs recorded from the right and left leg muscles (two-way ANOVA, *P*>0.05 for all parameters), suggesting that recruitment of neuronal elements for MEPs and TEPs was conducted in a similar order.

**Table 1:** Sigmoid function parameters.

	maximum area	R2	m	S50	slope	stimulus at threshold	stimulus at maximum
**Motor evoked potentials (MEPs)**							
RTA	4.91±0.65	0.94±0.02	6.18±1.05	1.59±0.27	0.40±0.07	1.19±0.22	1.98±0.33
RMG	0.88±0.18	0.93±0.02	5.34±0.54	1.73±0.28	0.39±0.04	1.33±0.25	2.12±0.31
RSOL	1.34±0.15	0.03±0.02	5.72±0.84	1.43±0.10	0.40±0.07	1.04±0.09	1.83±0.14
RPL	1.99±0.55	0.94±0.02	6.23±1.20	1.28±0.10	0.37±0.06	0.91±0.06	1.64±0.16
**Transpinal evoked potentials (TEPs)**							
RTA	3.81±1.11	0.94±0.02	4.42±0.63	1.45±0.20	0.46±0.07	0.98±0.13	1.91±0.26
RMG	5.05±0.03	0.97±0.01	6.54±0.21	1.49±0.20	0.31±0.01	1.18±0.19	1.80±0.21
RSOL	9.02±1.46	0.99±0.01	6.82±0.34	1.42±0.20	0.29±0.01	1.12±0.21	1.71±0.18
RPL	3.62±0.44	0.97±0.01	6.54±0.21	1.49±0.20	0.31±0.01	1.18±0.19	1.80±0.21
LTA	3.77±1.87	0.99±0.01	4.23±2.09	2.27±1.00	0.63±0.31	1.64±0.69	2.89±1.30
LMG	2.90±1.08	0.97±0.02	7.78±2.93	1.24±0.04	0.30±0.11	0.94±0.08	1.53±0.15
LSOL	5.19±3.00	0.98±0.01	5.66±1.58	1.41±0.18	0.38±0.11	1.02±0.07	1.79±0.28
LPL	2.96±1.11	0.97±0.01	7.30±3.16	1.30±0.33	0.34±0.15	0.96±0.18	1.63±0.47

Summarized sigmoid function parameters calculated from the sigmoid fit applied to the MEP and TEP recruitment curve for each subject and muscle. The first column indicates the maximum size of the action potentials from the recruitment curve (in mV ms), while the second column indicates the R^2^ from the sigmoid function. No statistically significant differences were found between the sigmoidal parameters between MEPs and TEPs, across muscles, or between TEPs recorded from the left or right leg (*P*>0.05). m: slope parameter of the function, S50: stimulus at 50% of the maximal evoked compound action muscle potential. R: right. L: left. TA: tibialis anterior. MG: medialis gastrocnemius. SOL: soleus. PL: peroneus longus.

### Effects of tsESS over the thoracolumbar region on ankle MEPs

**Figure 3:**
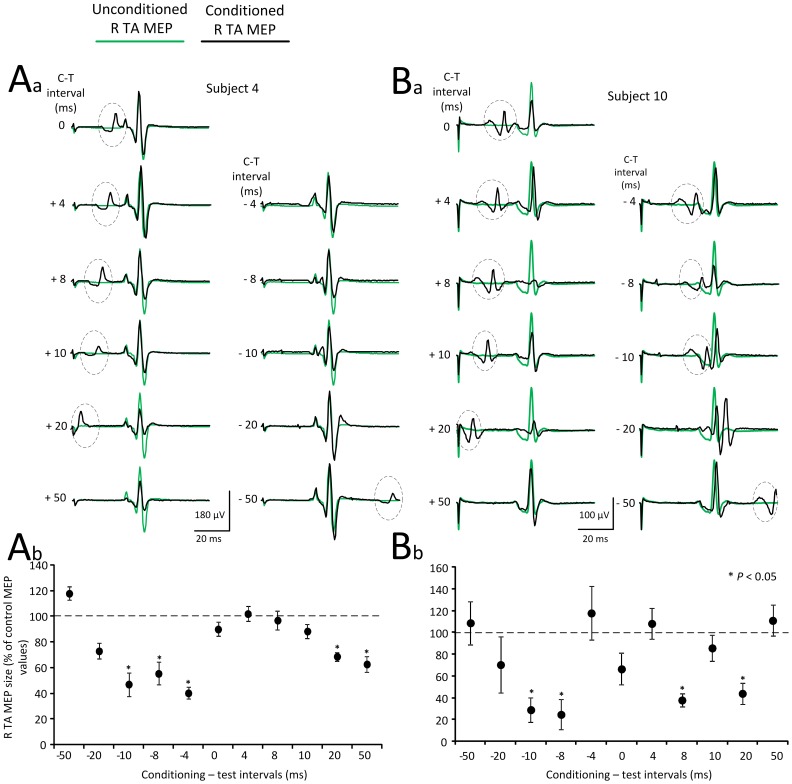
Effects of transcutaneous electric stimulation of the spine (tsESS) on TA MEPs. (**Aa, Ba**) Waveform averages of the right TA MEPs from two representative subjects under control conditions (green lines) and following tsESS (black lines) for all conditioning-test (C-T) intervals tested. The action potential within the dotted circle identifies the right TA TEP induced by the conditioning tsESS stimuli. All EMGs are shown as captured and subtraction to counteract summation of MEPs and TEPs was not applied. (**Ab, Bb**) Overall mean amplitude of the conditioned right TA MEPs for the same subjects (subjects 4 and 10), in which the net conditioning stimulus effect (i.e., the TEPs induced by the conditioning tsESS were subtracted) is indicated. Asterisks indicate statistically significant differences of conditioned MEPs from control values (*P*<0.05; one-way ANOVA). Error bars denote the SEM. TA: tibialis anterior. MEPs: motor evoked potentials. TEPs: transpinal evoked potentials. tsESS: transcutaneous electric stimulation of the spine.

The TA MEP resting threshold ranged from 39 to 79% of the maximum stimulator output (51.73±12.02; mean ± SD) across subjects. Control and conditioned ankle MEPs were recorded with TMS set at 1.2× TA MEP resting threshold. In Figures 3Aa and 3Ba, waveform averages of TA MEPs recorded from the right leg (contralateral to TMS) under control conditions (green lines) and following tsESS of the thoracolumbar region (black lines) at positive and negative C-T intervals are indicated for two subjects. TA MEP waveform averages are shown as depicted on the surface EMG, without the TEPs subtracted from MEPs at the C-T intervals that spinal summation between MEPs and TEPs was evident. For each subject, the overall amplitude of the normalized subtracted TA MEP is indicated in Figures 3Ab and 3Bb, respectively. In subject 4, tsESS reduced significantly the right TA MEP amplitude at the negative C-T intervals of 10, 8, and 4 ms and then again at the positive C-T intervals of 20 and 50 ms (Figure 3Ab; F_11_ = 76.31, *P*<0.001; one-way ANOVA). A short-latency TA MEP depression was also evident in subject 10 at the negative C-T intervals of 10 and 8 ms (Figure 3Bb) while no statistically significant differences were observed at the C-T intervals of −4, 0, and 4 ms. This was followed by depression of the R TA MEPs at the positive C-T intervals of 8 and 20 ms (Figure 3Bb; F_11_ = 46.26, *P*<0.001, one-way ANOVA).

**Figure 4:**
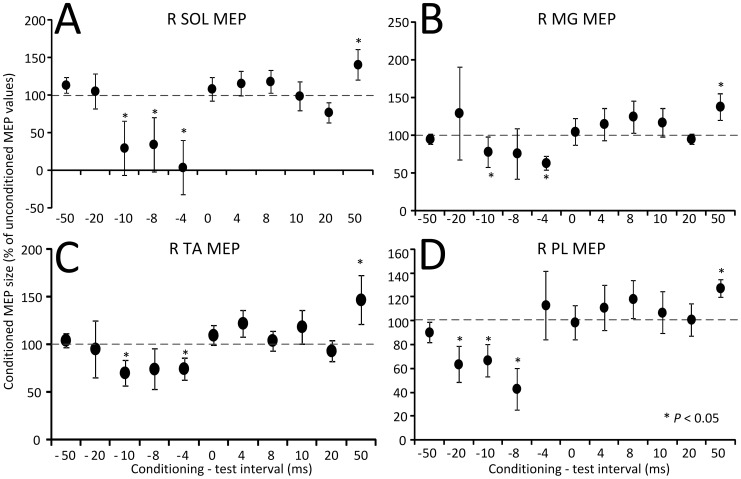
Effects of noninvasive transpinal stimulation on MEPs. Amplitude of MEPs recorded from the right (R) soleus (SOL), medialis gastrocnemius (MG), tibialis anterior (TA), and peroneus longus (PL) muscles following transcutaneous electric stimulation of the spine over the thoracolumbar region from 14 subjects. On the abscissa the conditioning-test interval (ms) tested is indicated. A negative C-T interval denotes that transcutaneous electric stimulation of the spine was delivered before TMS. Asterisks indicate statistically significant differences of conditioned MEPs from control values (*P*<0.05; one-way ANOVA). Error bars denote the SEM.

In Figure 4, the amplitude of the conditioned right SOL, MG, TA, and PL MEPs recorded from 14 subjects following tsESS is indicated. The C-T interval is denoted on the abscissa and the conditioned MEPs are presented as a percentage of the unconditioned associated MEP values. Kruskal-Wallis one-way ANOVA showed that the SOL MEPs varied significantly across the C-T intervals tested, with SOL MEPs at the C-T intervals of −10, −8, and −4 ms to be statistically significant different from control MEP values, while at the C-T interval of 50 ms the SOL MEP amplitude was increased compared to control MEP values (F_10_ = 30.87, *P*<0.001; Figure 4A). A similar result was also found for the right MG MEPs (F_10_ = 29.22, *P* = 0.011; Figure 4B). Likewise, the conditioned TA MEPs (F_10_ = 19.1, *P* = 0.039; Figure 4C) and PL MEPs (F_10_ = 2.61, *P* = 0.012; Figure 4D) varied significantly across the C-T intervals tested, and were depressed at negative C-T intervals and facilitated at the C-T interval of 50 ms. It is apparent that the effects of tsESS depend largely on the timing between TMS and tsESS, resulting in short-latency MEP depression followed by long-latency MEP facilitation.

### Effects of subthreshold TMS on TEPs

**Figure 5:**
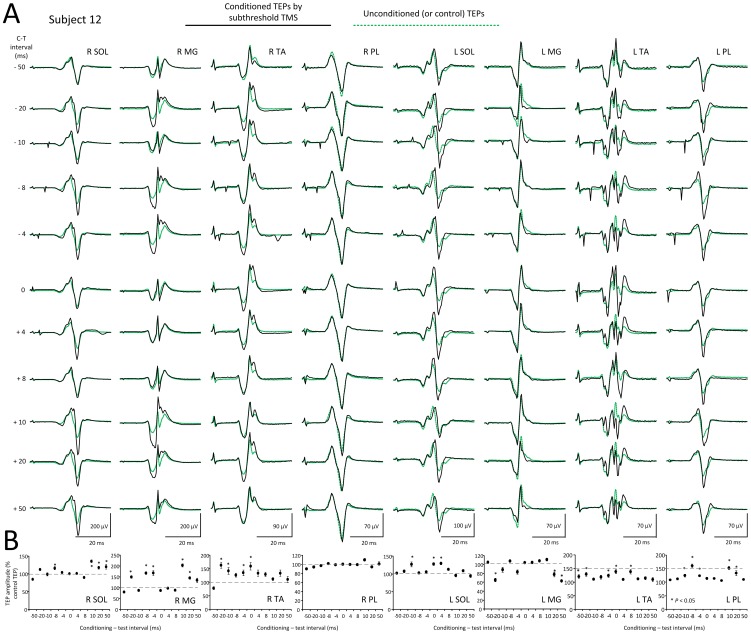
Effects of subthreshold TMS on TEPs. (**A**) Waveform averages of the right (R) and left (L) soleus (SOL), medialis gastrocnemius (MG), tibialis anterior (TA), and peroneus longus (PL) TEPs from one subject under control conditions (green lines) and following subthreshold TMS (black lines) for all conditioning-test (C-T) intervals tested. (**B**) Overall mean amplitude of the conditioned TEPs for the same subject. Asterisks indicate statistically significant differences of conditioned TEPs from control values (*P*<0.05; one-way ANOVA). Error bars denote the SEM. TEPs: transpinal evoked potentials. tsESS: transcutaneous electric stimulation of the spine. TMS: transcranial magnetic stimulation.

In Figure 5A, waveform averages of TEPs recorded from the right and left SOL, MG, TA, and PL muscles following subthreshold TMS delivered to the left M1 are indicated from one subject (subject 12) for all C-T intervals tested. TEPs under control conditions are shown as green dotted lines, while TEPs following subthreshold TMS are indicated as solid black lines. The normalized size of each conditioned TEP is indicated in Figure 5B. TEPs were either facilitated or remained unaltered following subthreshold TMS regardless the muscle or leg side from which they were recorded. For example, the right TA TEPs were increased at the negative C-T intervals, similar to that observed for the left TA TEPs (ipsilateral to TMS) (Figure 5B). Further, in this subject while the right MG TEPs were facilitated following subthreshold TMS, the left MG TEPs at these intervals were depressed.

**Figure 6:**
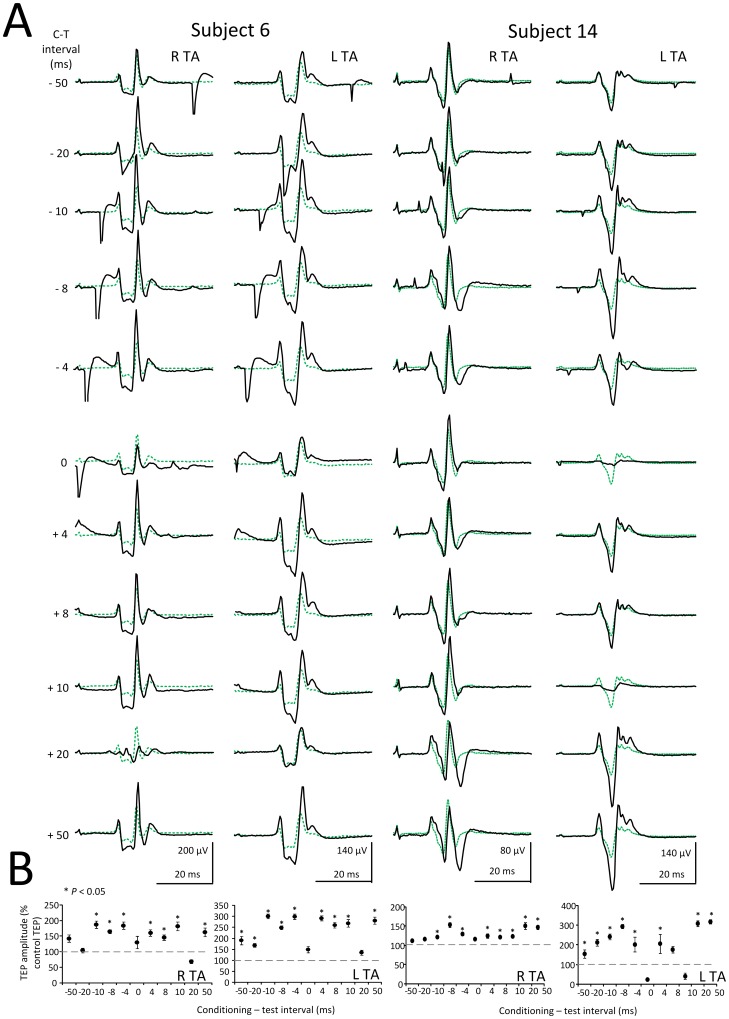
Effects of subthreshold TMS on right and left TA TEPs. (**A**) Waveform averages of the right (R) and left (L) TA TEPs in two additional subjects (subject 6 and subject 14) under control conditions (green dotted lines) and following subthreshold TMS (solid black lines) for all conditioning-test (C-T) intervals tested. (**B**) Overall mean amplitude of the conditioned TEPs for the same subjects. Asterisks indicate statistically significant differences of conditioned TEPs from control values (*P*<0.05; one-way ANOVA). Error bars denote the SEM.

To further demonstrate the effects of subthreshold TMS of the left M1 on ipsilateral and contralateral TA TEPs, waveform averages of right and left conditioned TA TEPs are indicated for two additional subjects (subjects 6 and 14) in Figure 6A, while the corresponding TEP amplitude as a percentage of the unconditioned associated TEP is presented in Figure 6B. In both subjects, subthreshold TMS delivered to the left M1 increased the left TA TEPs (ipsilateral to TMS) more compared to the right TA TEPs (contralateral to TMS) (Figure 6B), supporting that subthreshold TMS can change the amplitude of spinal potentials of both legs.

**Figure 7:**
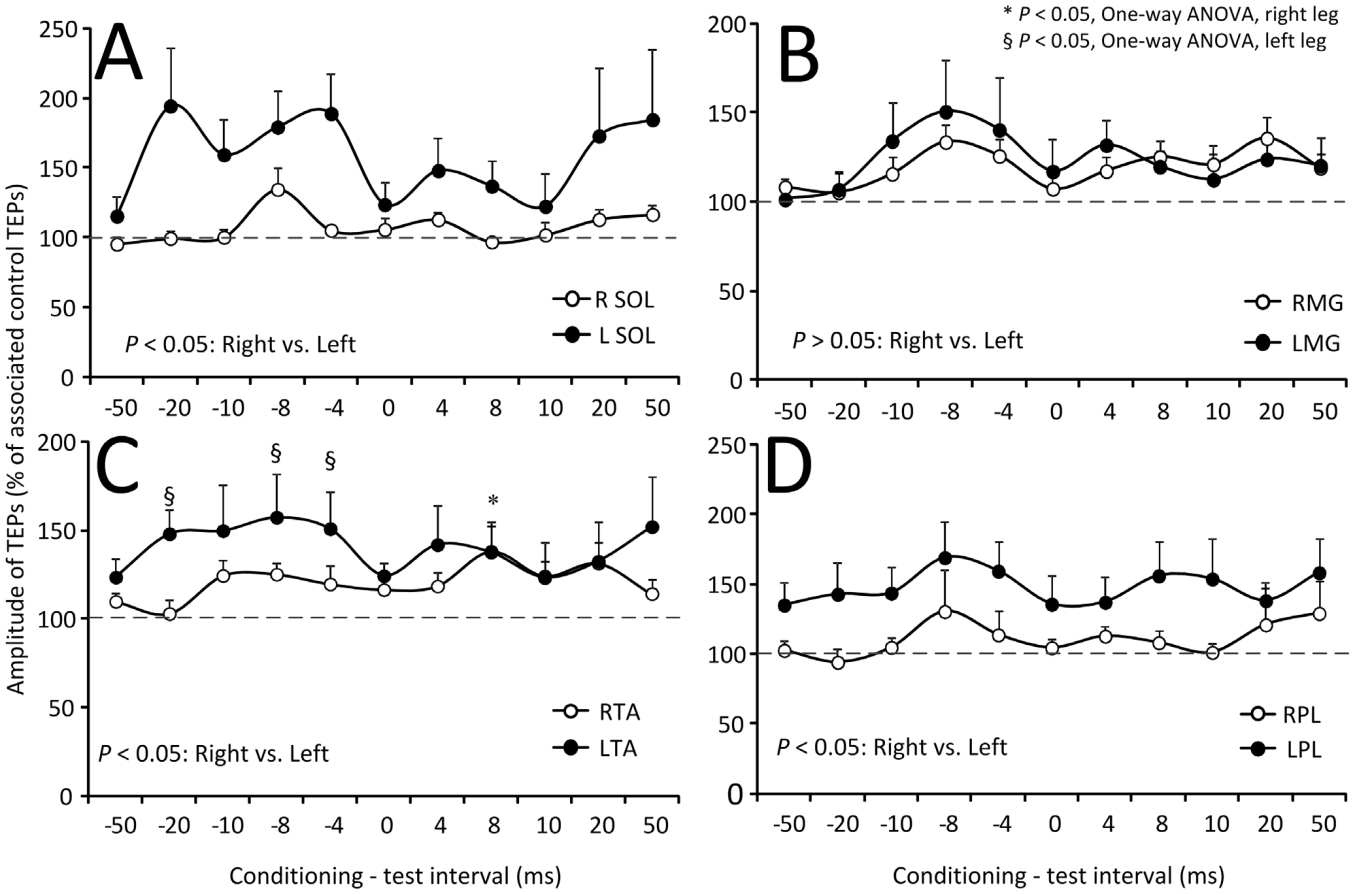
Modulation of TEPs by subthreshold TMS. Overall mean amplitude of TEPs recorded from the right (R) and left (L) soleus (SOL), medialis gastrocnemius (MG), tibialis anterior (TA), and peroneus longus (PL) muscles following transcranial magnetic stimulation (TMS) delivered at intensities that motor evoked potentials were not evoked. On the abscissa the conditioning-test (C-T) interval (ms) is indicated. A negative C-T interval denotes that TMS was delivered after transcutaneous electric stimulation of the spine. Symbols “*” and “§” denote statistically significant differences of conditioned TEPs from control values for the right or left side TEPs, respectively. Error bars denote the SEM.

The amplitude of the conditioned SOL, MG, TA, and PL TEPs recorded from 14 subjects and both legs following subthreshold TMS is presented in Figure 7. The C-T interval is denoted on the abscissa while the conditioned TEPs are presented as a percentage of the mean amplitude of the unconditioned TEPs. Kruskal-Wallis one-way ANOVA on ranks showed that the right and left SOL TEPs did not vary significantly across the C-T intervals tested (R SOL: F_11_ = 5.76, *P* = 0.889; L SOL: F_11_ = 5.76, *P* = 0.5) (Figure 7A). This result was also found for TEPs recorded from the right and left MG and PL muscles. In contrast, the right and left TA TEPs varied significantly across the C-T intervals tested (F_11_ = 34.37, *P*<0.001) (Figure 7C). Kruskal-Wallis two-way ANOVA showed that the conditioned SOL TEPs amplitude was statistically significant different between the right and left legs (F = 26.6, *P*<0.001). The same result was also found for the TA TEPs (F = 10.43, *P* = 0.001) and for the PL TEPs (F = 17.44, *P*<0.001), suggesting that TEPs ipsilateral to TMS were facilitated more compared to TEPs contralateral to TMS.

### Effects of suprathreshold TMS on TEPs

**Figure 8:**
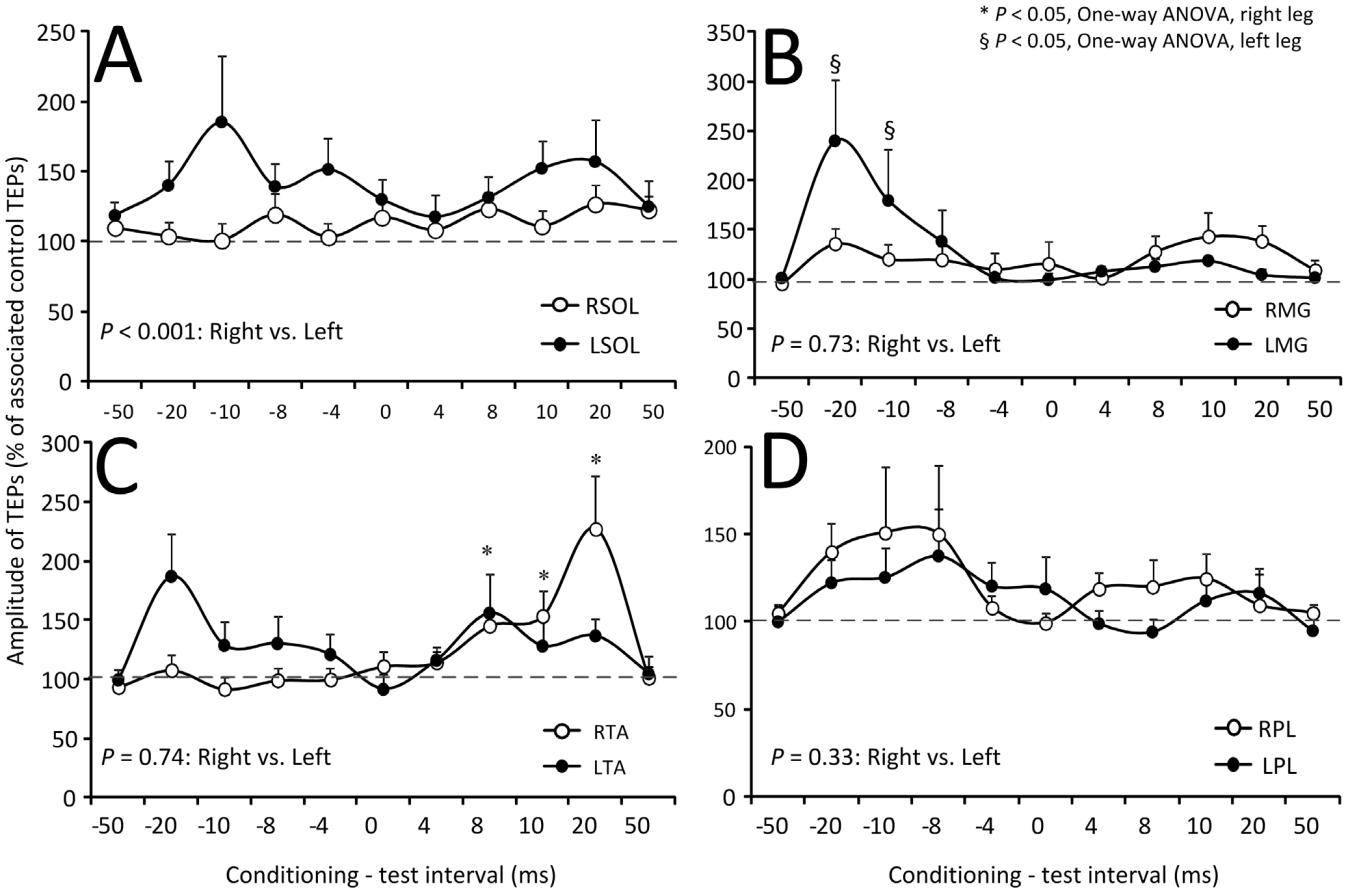
Modulation of TEPs by suprathreshold TMS. Overall mean amplitude of TEPs recorded from the right (R) and left (L) soleus (SOL), medialis gastrocnemius (MG), tibialis anterior (TA), and peroneus longus (PL) muscles following transcranial magnetic stimulation (TMS) delivered above TA MEP resting threshold. On the abscissa the conditioning-test (C-T) interval (ms) is indicated. A negative C-T interval denotes that TMS was delivered after transcutaneous electric stimulation of the spine. Symbols “*” and “§” denote statistically significant differences of conditioned TEPs from control values for the right or left side TEPs, respectively. Error bars denote the SEM.

The amplitude of the conditioned SOL, MG, TA, and PL TEPs recorded from 14 subjects and both legs following suprathreshold TMS is presented in Figure 8. The C-T interval is denoted on the abscissa while the conditioned TEPs are presented as a percentage of the associated unconditioned TEPs. Kruskal-Wallis one-way ANOVA on ranks showed that the right and left SOL TEPs did not vary significantly across the C-T intervals tested (R SOL: F_11_ = 5.76, *P* = 0.889; L SOL: F_11_ = 10.23, *P* = 0.5) (Figure 8A). This result was also found for TEPs recorded from the right and left MG and PL muscles. In contrast, the right and left TA TEPs varied significantly across the C-T intervals tested (F_11_ = 34.37, *P*<0.001) (Figure 8C). Kruskal-Wallis two-way ANOVA showed that the conditioned SOL TEPs amplitude was statistically significant different between the right and left legs (F = 11.3, *P*<0.001) but not statistically significant different between the left and right TA TEPs (F = 0.11, *P* = 0.74), MG TEPs (F = 0.11, *P* = 0.73), or PL TEPs (F = 0.93, *P* = 0.33).

ANOVA for repeated measures showed that the difference in mean values of TA TEPs varied significantly among C-T intervals (F_10_ = 2.7, *P* = 0.002) and leg side (F_1_ = 5.8, *P* = 0.01), while the effects at different C-T intervals depended on the TMS intensity (F_10_ = 2.1, *P* = 0.018) and on the leg side (F_10_ = 2.4, *P* = 0.009). Similarly, the PL TEPs amplitude was different with respect to the TMS intensity (F_1_ = 5.01, *P* = 0.026), and depended on the leg side from which the TEPs were recorded (F_1_ = 15.2, *P*<0.001). The SOL and MG TEPs among different levels of TMS intensity was not statistically significant different (SOL: F_1_ = 0.77, *P* = 0.38; MG: F_1_ = 0.2, *P* = 0.65), but a statistically significant difference among the right and left legs for the SOL TEPs (F_1_ = 40.21, *P*<0.001) and among the different C-T intervals for the MG TEPs (F_10_ = 2.08, *P* = 0.02) was found.

## Discussion

This work demonstrated that noninvasive transpinal and transcortical stimulation alters corticospinal excitability and spinal motor output in healthy humans. Specifically, we demonstrated that MEPs and TEPs have similar recruitment characteristics and are summated at a spinal level. Further, noninvasive transpinal stimulation induced short-latency MEP depression followed by long-latency MEP facilitation of ankle plantar flexors and extensors. Last, subthreshold TMS increased spinal motor output more in the leg ipsilateral to TMS, while suprathreshold TMS induced long-latency facilitation of ankle flexor TEPs and short-latency facilitation of ankle extensor TEPs.

### On the neurophysiological properties of TEPs and MEPs

The sigmoidal nature of MEPs and TEPs input-output relation (Figure 2) is consistent to the well-known recruitment order of motor axons and group I afferents mediating monosynaptic reflexes [16], [23]. The MEP recruitment curves recorded from ankle plantar flexors and extensors had a similar shape to that previously reported [23], while the shape of the TEP recruitment curves resembled the one recorded following cathodal tsESS at Thoracic 11/12 to Sacral 1/2 [24]. The sigmoidal input-output curve reflects the well established fact that stimuli of increasing strength recruit motor axons with increasing motor unit potentials with a wide distribution of spike amplitudes [25]. Thus, the similar shape and sigmoid function parameters for MEP and TEP recruitment curves (Figure 2 and Table 1), signify that recruitment of corticospinal elements following TMS and spinal elements following tsESS was conducted in a similar order, and likely channeled through common neuronal pathways. The latter is supported by the summation of the right TA TEPs and MEPs at a spinal level when tsESS and TMS were delivered above response threshold level (Figure 1). Summation of action potentials was evident when TMS was delivered 8, 10, and 20 ms before tsESS in cases that tsESS was the conditioning stimuli, and when TMS was delivered 8, 10, and 20 ms after tsESS in cases that TMS was the conditioning stimulus. Our findings are consistent with the summation of the soleus MEPs and TEPs reported following paired cathodal tsESS and TMS during voluntary plantarflexion in healthy humans [9]. The summation of TEPs and MEPs reflects addition of action potentials from different afferent groups but most importantly that tsESS and TMS may excite the same group of motor units and thus the same motoneurons from the pool. Nonetheless, peristimulus time histograms of single motor unit recordings for TEPs and MEPs of similar sizes and experiments that can delineate the exact neuronal pathways and cortico-motoneuronal and cortico-interneuronal circuits activated by TMS and tsESS are needed.

At this point, additional neurophysiological properties of TEPs and MEPs need to be considered. Because TEPs and MEPs recorded from different muscles do not have similar latencies and shapes, we may theorize that they are associated with different synaptic events and thus have a different origin. Further, presynaptic inhibition of corticospinal volleys is absent in both animals and humans [26], [27]. Depression of orthodromic Ia afferent transmission with increasing stimulus repetition rates that occurs at a presynaptic level is well established in humans [2]. This depression was absent in arm or leg TEPs following electric or magnetic stimulation of the spine in all but one subject in the arm TEPs [10]–[12], consistent to the finding that homosynaptic depression depends on the type of afferents and the target neurons [28]. Paired tsESS stimuli reduce significantly the amplitude of the second TEP [9], [29]. Based on this phenomenon, TEPs have been regarded equivalent to H-reflexes [7]. However, depression due to paired stimuli delivered at a constant stimulation rate cannot be attributed to synaptic events associated with homosynaptic depression of Ia afferent transmission [30]. In addition, the decreased in amplitude responses upon double stimuli cannot easily be termed as short-latency reflexes since the soleus H-reflex is facilitated when double stimuli are delivered at interstimulus intervals that range from 25 to 100 ms [31], [32]. Interestingly, suppressive interactions following paired stimuli were reported when stimulation was delivered to the ventral side and facilitatory when stimulation was delivered to the dorsal surface and intraspinal sites [33]. Further, peristimulus time histograms of single motor units following stimulation of the spine with an implanted electrode in the epidural space showed that spinal cord stimulation activates antidromically Ia afferents that in turn results in monosynaptic facilitation of motoneurons and reduction in transmission of reciprocal Ia inhibition [5], consistent to the excitation site suggested following electric or magnetic stimulation of the spine [34], [35]. Collectively, TEPs may not be reflexly-mediated action potentials in the conventional known manner but constitute composite excitatory potentials of motor nerve fibers excited orthodromically and different types of afferents excited antidromically in the majority of subjects [10]–[12]. This means that TEP excitability may be used as a diagnostic tool in neurological disorders bypassing the hyperexcitable spinal alpha motoneurons. Based on the above discussed evidence, it is clear that further research on the physiological differences and similarities of these action potentials is needed.

### Corticospinal (MEPs) excitability following transpinal stimulation

Transcutaneous electric stimulation of the spine over the thoracolumbar region decreased significantly MEP excitability in seated semi-prone subjects at negative C-T intervals, followed by long-latency MEP facilitation in ankle plantar flexors and extensors (Figure 4), supporting that depending on the time of arrival of action potentials induced by TMS and tsESS, corticospinal excitability can increase or decrease.

TMS of M1 evokes several volleys of corticospinal activity at the spinal level [36]–[38]. The earliest direct (D) - waves originate from axonal activation of corticospinal neurons, while the later indirect (I) - waves result from indirect activation of corticospinal neurons via cortical interneurons [39]–[43]. These waves have a conduction velocity of 62–66 m/s and appear within 2.0 to 4.0 ms at the cervical and thoracic spinal levels with a refractory period of 2 ms [37], [44]. Consequently, at the negative C-T interval of 10, 8, and 4 ms (tsESS delivered after TMS), corticospinal volleys had ample time to reach the thoracolumbar spinal cord and be affected by the conditioning tsESS stimuli. Additionally, hyperpolarization of motoneurons by the transpinal conditioning stimuli may have contributed to the short-latency MEP depression.

tsESS at longer C-T intervals induced a significant facilitation of MEPs recorded from ankle plantar flexors and extensors (Figure 4). The tsESS-induced long-latency MEP facilitation is consistent with the long-latency soleus and TA MEP facilitation following tibial or common peroneal nerve stimulation [45], [46], with the facilitation of somatosensory evoked potentials following cathodal direct current stimulation delivered at the thoracic level in anaesthetized animals [47], and with the facilitation of the TA EMG responses at longer latencies following cathodal tsESS over the thoracolumbar region [9]. At these long C-T intervals, group I afferent volleys of paraspinal muscles can arrive at the somatosensory cortex and affect corticospinal excitability through transcortical circuits [48], since group I afferent volleys decrease short-latency intracortical inhibition and increase intracortical facilitation in the M1 region controlling the TA muscle [49]. Last, because the effect was similar in MEPs recorded from ankle plantar flexors and extensors, it is likely that the short-latency MEP depression and long-latency MEP facilitation were mediated by similar neuronal pathways for different alpha motoneurons.

Changes in MEP amplitude have been reported during shortening or lengthening muscle contractions, before the onset of ankle joint movement, during walking, and following repetitive stimulation of the common peroneal nerve [13], [50]–[52]. Because changes in MEP amplitude reflect a change in membrane excitability of pyramidal or excitatory interneurons or a change in the synaptic efficacy between cortical neurons, and MEP amplitude is sensitive to the excitability state of spinal motoneurons and interneurons [53], [54], it is inappropriate to assign the observed effects to a specific neuronal pathway. However, given that the increased MEPs at the C-T interval of 50 ms following peripheral nerve stimulation were mediated largely by cortical facilitatory mechanisms [49], it is likely that MEPs at this long-latency were affected simultaneously at cortical, subcortical, and spinal levels.

### Spinal output (TEPs) following transcortical stimulation

In contrast to the bimodal MEP modulation pattern by transpinal stimulation, TEPs were facilitated by subthreshold and suprathreshold TMS (Figures 5–8). These findings have never been documented in humans and have great clinical significance because TMS and tsESS can be utilized as a modality to increase spinal motor output in neurological cases.

Subthreshold TMS over the left M1 increased more the TEPs recorded from muscles ipsilateral to TMS compared to TEPs recorded from muscles contralateral to TMS (Figure 7). TMS, delivered at intensities that do not induce descending motor volleys and direct motoneuron discharges, can influence corticospinal and spinal output through intracortical inhibitory and facilitatory cells [42], [55]. For example, subthreshold TMS suppresses the ongoing EMG and motoneuron activity, and induces short-latency facilitation of the soleus H-reflex [56], [57]. Because the effects in this study were observed at the negative C-T intervals of 4, 8, and 20 ms (Figure 7C), it is possible that subthreshold TMS activated primarily intracortical facilitatory neuronal pathways potentiating depolarization of TA motoneurons.

Suprathreshold TMS increased TEPs amplitude differently from that observed following subthreshold TMS. Specifically, the right PL TEPs were increased more with suprathreshold TMS than with subthreshold TMS (Figure 7D, 8D), while the left TA TEPs facilitation at short-latencies following subthreshold TMS (Figure 7C) occurred at longer latencies (at C-T intervals that summation of MEPs and TEPs was evident) following suprathreshold TMS (Figure 8C). It should be noted that summation at the longer intervals was counteracted and thus the TEP sizes show a net facilitation by suprathreshold TMS. Suprathreshold TMS may increase TEPs amplitude through spatial distribution of D- and I-waves in the spinal cord [58], spatiotemporal summation of action potentials induced by the conditioning stimuli potentiating the depolarization of alpha motoneurons, and reduced actions of reciprocal inhibitory interneurons due to TMS and tsESS [5]. Last, because the left MG TEPs facilitation by suprathreshold TMS occurred at short latencies during which summation between MEPs and TEPs did not occur (Figure 8B), transcortical stimulation increases TEPs amplitude regardless the function of motoneurons (flexors vs. extensors).

### Functional considerations

The biophysical properties of electromagnetic stimulation of M1 and spine need to be considered. It is well known that the magnitude of the cortical current density is influenced by the type of the magnetic coil, the relative coil-to-tissue distance, and by the conductivity, heterogeneity, and anisotropy of the neural tissue [59]–[61]. Phantom model recordings, imaging studies, deep electrode recordings, and electromagnetic simulation models have shown that the focality of magnetic stimulation delivered with a figure-of-eight coil at 90 to 100% of the maximum stimulator output is approximately 25 mm^2^, the area of stimulation is 100–200 mm^2^, and the strength of the magnetic field lowers at 25 mm below the coil surface [61], [62]. Similarly, the double cone coil has a focal area of 94 mm^2^ [63], and induces a more deeply penetrating and less focal electric field compared to figure-8 coil [64]. Further, transcutaneous stimulation over the spine generates action potentials in neural tissue with a depth of 5 cm [65]. In addition to these factors, a difference between TMS and the biological effect (MEP) can only be accurately related through a stimulus localization method (fMRI) and stereotaxic-navigational systems [66]. Taken altogether, the electrical field induced following TMS and electric stimulation of the spine warrant further investigation. This can be addressed in future studies in which physiological findings (MEPs and TEPs) are studied along with stereotaxic anatomical measurements. Such findings may contribute to the development of transpinal and transcortical stimulation protocols based on an in-depth understanding of the underlying physiological mechanisms and anatomical sites.

## Conclusions

This study showed that tsESS of the thoracolumbar region induced short-latency MEP depression followed by long-latency MEP facilitation, and when tsESS was combined with subthreshold or suprathreshold TMS spinal motor output was facilitated. Based on our current and published findings [10]–[12], tsESS can be utilized in upper motor neuron lesions to normalize reflex hyper-excitability of upper and lower limbs, increase motor output of many spinal segments, and alter corticospinal excitability.
